# P-2031. Thorough QT/QTc Clinical Study to Evaluate the Effect of Remdesivir on Cardiac Repolarization in Healthy Participants

**DOI:** 10.1093/ofid/ofae631.2187

**Published:** 2025-01-29

**Authors:** Olena Anoshchenko, Mazin Abdelghany, Robert H Hyland, Santosh Davies, Anna Kwan, Ran Duan, Aryun Kim

**Affiliations:** Gilead Sciences, Inc., Foster City, California; Gilead Sciences, Inc., Foster City, California; Gilead Sciences, Inc., Foster City, California; Gilead Sciences, Inc., Foster City, California; Gilead Sciences, Inc., Foster City, California; Gilead Sciences, Inc., Foster City, California; Gilead Sciences, Inc., Foster City, California

## Abstract

**Background:**

Remdesivir (RDV) is an intravenous nucleotide prodrug approved for COVID-19 treatment. Its effect on QT interval is unknown. This Phase 1 study evaluated the effects of a supratherapeutic dose of RDV on QT interval.

**Figure 1.**
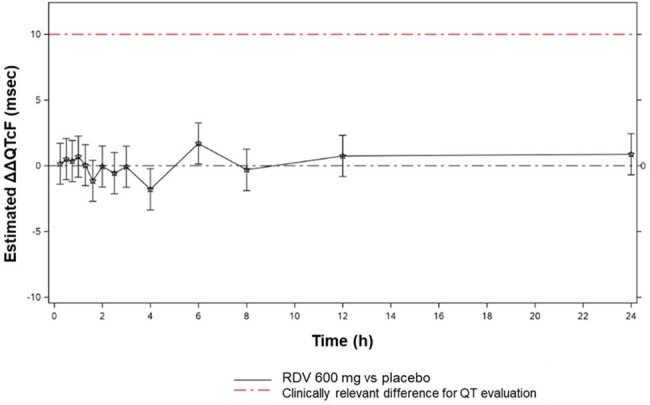
Estimated ΔΔQTcF (90% CI) between RDV and Placebo-to-match RDV. ΔΔQTcF, baseline-adjusted, placebo-matched QTcF; QTcF, QT interval corrected for heart rate using the Fridericia formula; RDV, remdesivir.

**Methods:**

This study included 2 cohorts of healthy participants aged 18-55 years: an RDV dose-selection cohort (Sentinel Cohort; n=6); and a randomized, placebo- and positive-controlled (moxifloxacin), single-dose crossover cohort (Thorough QT [TQT] Cohort; n=55). The Sentinel Cohort assessed pharmacokinetics (PK) and safety of a supratherapeutic RDV dose (600 mg) and was followed by the TQT Cohort. The primary endpoint for the TQT Cohort was ΔΔQTcF: the difference between baseline-adjusted QTcF (QT corrected for heart rate [HR] using the Fridericia formula) in the RDV treatment period and baseline-adjusted QTcF in the placebo period at each postdose time point. Adverse events (AEs), laboratory abnormalities, and plasma PK were also assessed.

**Table 1.**
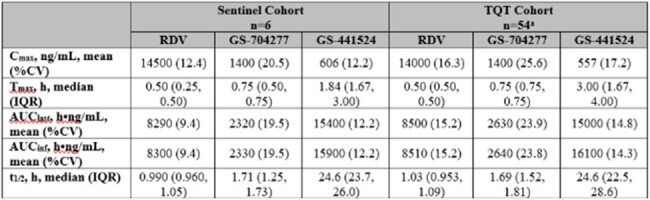
Plasma PK Parameters for RDV and Its Metabolites (GS-704277 and GS 441524) Following a Single Dose of RDV 600 mg. AUCinf, area under the concentration-time curve extrapolated to infinite time; AUClast, area under the concentration-time curve from dosing to last measurable concentration; Cmax, maximum observed concentration; CV, coefficient of variance; PK, pharmacokinetic; RDV, remdesivir; t½, half-life; Tmax, time at which maximum concentration was observed; TQT, thorough QT.

aThe TQT Cohort enrolled 55 participants; 54 participants received RDV.

**Results:**

Following RDV 600 mg, PK exposures of RDV and its metabolites were similar in both cohorts (Table 1). In the TQT Cohort, the assay sensitivity was established; the estimated lower bound of 96.67% CI for ΔΔQTcF for moxifloxacin was >5 msec for ≥1 of the prespecified 1, 2, and 3h postdose time points. RDV had no clinically relevant effects on QT interval compared to placebo (Figure 1); the estimated upper bound of 90% CI for RDV ΔΔQTcF was < 10 msec at all postdose time points. Most AEs were Grade 1-2 in severity. One participant receiving RDV in the TQT Cohort had a Grade 3 AE (transaminase increased). There were no other Grade ≥3 AEs, tachycardia, or HR AEs (Table 2). 5/54 participants in the TQT Cohort had HR >100 bpm within 12h of receiving RDV (all resolved by 24h postdose). 3 of the 5 participants had Grade 1-2 nausea and vomiting ≤13h postdose; 1 of the 3 participants had treatment-related chills ≤7h postdose.

**Table 2.**
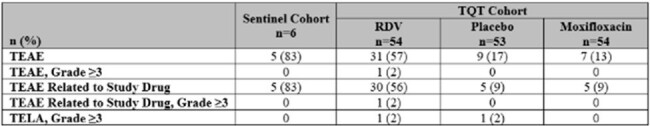
TEAEs and Grade ≥3 TELAs TEAE, treatment-emergent adverse event; TELA, treatment-emergent laboratory abnormality; RDV, remdesivir; TQT, thorough QT.

**Conclusion:**

RDV 600 mg in healthy participants resulted in PK exposures 3-4–fold higher than those observed with RDV 200 mg in prior studies. A single dose of RDV 600 mg was generally safe; there were no AEs associated with QT prolongation, deaths, or SAEs reported. In this positive control–validated study, ΔΔQTcF for RDV was below the regulatory threshold of concern, demonstrating that its clinical regimen has no proarrhythmic potential.

**Disclosures:**

Olena Anoshchenko, PhD, Gilead Sciences, Inc.: Employee|Gilead Sciences, Inc.: Stocks/Bonds (Public Company) Mazin Abdelghany, MD, Gilead Sciences, Inc.: Employee|Gilead Sciences, Inc.: Stocks/Bonds (Public Company) Robert H. Hyland, DPhil, Gilead Sciences, Inc.: Employee|Gilead Sciences, Inc.: Stocks/Bonds (Public Company) Santosh Davies, MD, Gilead Sciences, Inc.: Employee|Gilead Sciences, Inc.: Stocks/Bonds (Public Company) Anna Kwan, BS, Gilead Sciences, Inc.: Employee|Gilead Sciences, Inc.: Stocks/Bonds (Public Company) Ran Duan, PhD, Gilead Sciences, Inc.: Employee|Gilead Sciences, Inc.: Stocks/Bonds (Public Company) Aryun Kim, PharmD, Gilead Sciences, Inc.: Employee|Gilead Sciences, Inc.: Stocks/Bonds (Public Company)

